# Possibilities of dissemination of specialist knowledge and acting capacity in the field of child protection in medicine: a qualitative survey

**DOI:** 10.3205/zma001303

**Published:** 2020-02-17

**Authors:** Anna Maier, Miriam Rassenhofer, Ulrike Hoffmann, Jörg M. Fegert

**Affiliations:** 1 Universityhospital of Ulm, Department of Child and Adolescent Psychiatry/Psychotherapy, Ulm, Germany

**Keywords:** health professionals, dissemination, continuous medical education, child abuse, qualitative research

## Abstract

**Objective: **The shortage of skilled workers and overloaded schedules make further training of health professionals difficult. In addition, child protection is not a systematic part of medical studies. The evaluation of an online course on child protection in medicine reveals positive feedback but also that the main reason for participants aborting the course is lack of time. Dissemination, as an active, targeted spreading of knowledge, can help to further spread knowledge about child protection in the target group. The aim of this article is to investigate whether and how the contents of the online course can be disseminated by professionals who have completed the online course.

**Methodology:** The data were collected through a quantitative online evaluation and qualitative telephone interviews with doctors who had completed the online course and evaluated it using an interpretive-reductive analysis.

**Results:** The respondents consider the need for further training and dissemination measures on the topic of child protection in medicine to be high. However, lack of time and insufficient relevance of the topic would present obstacles in the implementation of such measures. Meaningfulness and time off work or remuneration would in turn create incentives for implementation. Participants in dissemination measures could be motivated for example by further education points. In addition we were able to identify possible approaches for the implementation of such measures.

**Conclusion: **Various parameters influence the motivation of doctors regarding the implementation/perception of dissemination measures. Based on these, recommendations for action are given for different areas of the health care system, such as supplementing the training curricula and providing ready-made materials for dissemination.

## Zusammenfassung

**Zielsetzung: **Fachkräftemangel und zeitliche Überlastung erschweren die Fortbildung von Gesundheitsfachkräften. Kinderschutz ist zudem kein systematischer Bestandteil des Medizinstudiums. Die Evaluation eines Online-Kurses zu Kinderschutz in der Medizin zeigt dessen positive Beurteilung, aber auch, dass der Hauptgrund für den Abbruch der Kursbearbeitung Zeitmangel ist. Dissemination, als aktive zielgerichtete Verbreitung von Wissen, kann helfen, Wissen zu Kinderschutz weiter in der Zielgruppe zu verbreiten. Ziel dieses Beitrages ist es, zu untersuchen, ob und wie die Inhalte des Online-Kurses von Absolvent_innen disseminiert werden können.

**Methodik: **Die Daten wurden mittels einer quantitativen Online-Evaluation und qualitativer Telefoninterviews mit ärztlichen Absolvent_innen des Online-Kurses erhoben und mit einer interpretativ-reduktiven Analyse ausgewertet.

**Ergebnisse: **Der Bedarf an Fortbildungen und Disseminationsmaßnahmen zum Thema Kinderschutz in der Medizin wird von den Befragten als hoch angesehen. Zeitmangel, eine zu geringe Relevanz des Themas wären allerdings Hürden für die Durchführung solcher Maßnahmen. Die Sinnhaftigkeit, eine Freistellung von der Arbeit oder Entlohnung würde wiederrum Anreize zur Durchführung schaffen. Teilnehmende an Disseminationsmaßnahmen könnten, z.B. durch Fortbildungspunkte, motiviert werden. Des Weiteren konnten Ansätze für die Umsetzung solcher Maßnahmen ermittelt werden.

**Schlussfolgerung: **Verschiedene Parameter beeinflussen die Motivation von Ärzt_innen bzgl. Durchführung/Wahrnehmung von Disseminationsmaßnahmen. Basierend darauf werden Handlungsempfehlungen für verschiedene Bereiche des Gesundheitssystems gegeben, wie z.B. die Ergänzung der Ausbildungscurricula und das Angebot von vorgefertigten Disseminationsmaterialien.

## 1. Introduction

The shortage of skilled workers and the associated overloaded work schedules in medicine are a well-known and current topic [1], [2], [3]. In the medical field in particular, with constant new developments and insights, further training, which is associated however with additional time cost, is particularly important. In the area of medical child protection, too, there are and have been numerous new insights and legal changes, which are not well enough known, and which lead to uncertainty regarding the authority to act among health professionals [4]. The World Health Organization assumes that 90% of abuse cases in medical institutions go unnoticed [5]. 

Knowledge or education on the topic is an important tool in protecting children and adolescents from abuse [6]. It is therefore important to train as many health professionals as possible, as dangers to the welfare of children can emerge in all areas of medicine. The more widely essential knowledge is disseminated in the target group, the better (suspected) cases can be dealt with or the children and adolescents referred to the appropriate sources of support. The online course “Child protection in medicine – a basic course for all health professionals” sponsored by the Federal Ministry of Health of the Hospital for Child and Adolescent Psychiatry/Psychotherapy at the University Hospital Ulm addresses this problem [https://grundkurs.elearning-kinderschutz.de]. The course has a modular structure and contains learning units on various topics of child protection. In addition to imparting theoretical knowledge using basic and legal texts, special emphasis is placed on case-based learning and the development of practical action and emotional competencies and thus the reduction of operational uncertainties in the field of child protection. Participation in the course is free of charge during the promotional phase which runs until September 2020.

The pseudonymized evaluation by doctors who have completed the course shows a high level of satisfaction with the contents and its relevance for professional medical practice. It is therefore particularly important to put this potential to use. It also became clear that further training on the subject of child protection in medicine receives too little consideration in the eyes of doctors and that access to it must become more flexible. The most common reason for not completing the course was lack of time [7]. 

In addition to the classic transfer of knowledge, for example through specialist journals or external training events, the active and targeted dissemination of existing knowledge (dissemination) through colleagues, superiors etc. (multipliers) as part of the continuing education of health professionals is becoming increasingly important in overcoming the lack of time for training in the medical field [8], [9], [10].

The aim of this article is therefore to investigate to what extent professionals who have completed the online course can be used to effectively disseminate the learning contents of the online course to their colleagues. The focus here is on motivation and obstacles for future participants and multipliers, but also on concrete possibilities for implementation.

## 2. Methods

### 2.1. Recruitment of participants

**Figure 1:**
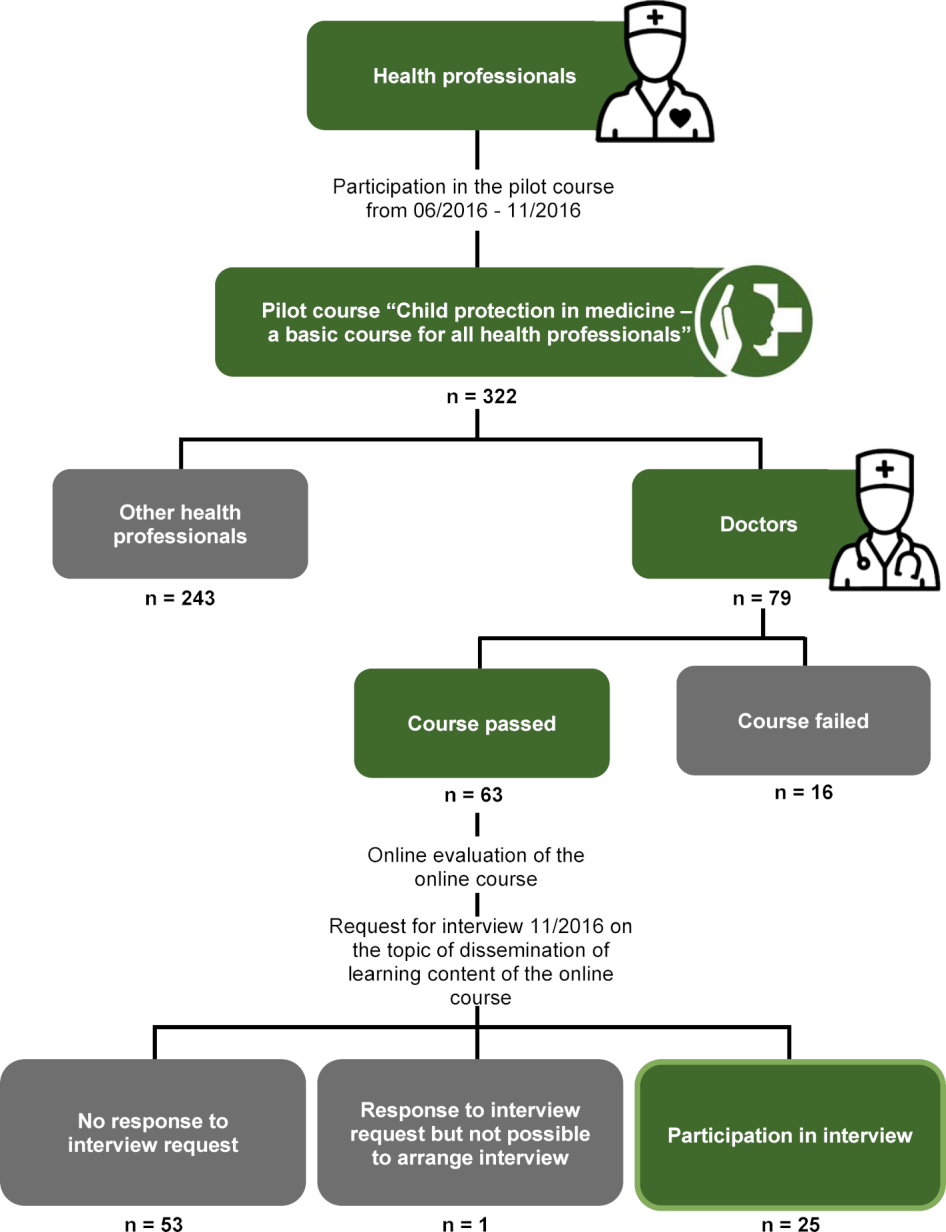
Recruitment of participants to evaluate the online course and the interview partners.

The prerequisites for participating in an interview was the successful completion of the pilot online course “Child protection in medicine - a basic course for all health professionals” from June to November 2016, in which all health professionals were able to participate; and a license to practice medicine. In November 2016, we write to all doctors who had completed the course to inquire if they would be willing to participate in a telephone interview. Interested parties were able to register online for a month and were then contacted with suggested dates for the interview (see figure 1 (Fig. 1)).

By participating in the interviews, the interviewees did not undertake to act as multipliers but merely to indicate an interest in doing so and were asked to respond from the perspective of a future multiplier during the interview.

The interviews were conducted in January and February 2017.

#### 2.2. Data collection

All participants who had completed the online course had to evaluate it in a quantitative online questionnaire. The questionnaire also asked whether and how the contents and materials of the online course had already been disseminated by the professionals who have completed the online course. The meaning of “dissemination of contents and materials from the online course” was explained to the evaluators beforehand.

In addition to the quantitative survey, in order to generate hypotheses on how the dissemination of content from the online course can be designed as effectively as possible, individual and detailed opinions on motivation, obstacles and implementation possibilities were obtained using a qualitative research design based on semi-structured, guideline-based telephone interviews. The structural and content-related design of the interview guide was discussed and developed based on specialist literature and the previous experience of the authors.

At the beginning of the interviews, demographic data were gathered and a stimulus opening question was asked. The interview guide included the areas of “Continuing education in the field of child protection in medicine”, “Dissemination of the learning contents of the online course” and “Activity as a multiplier” and asked about participation/carrying out a dissemination, obstacles and motivation separately. The interviews were each concluded with a summarizing question that aimed to stimulate the assessment of the topic [11], [12], [13], [14], [15] (see the “Guide” in the attachment 1 ).

A method sheet was developed [16], [17], [18] (see attachment 2 ) to make it easier for the interviewees to gain access to common methods of adult education, as no didactic skills are taught in the online course itself. This was emailed to the interviewees together with an information sheet in which, among other things, the goal of the interview was set out.

The interview was designed to last approximately 30 minutes.

#### 2.3. Transcription

With the consent of the interviewees the interviews were recorded using a dictation machine. The data were then anonymized and fully transcribed. Each interview transcript was then again compared with the original recordings in order to correct errors and define non-verbal aspects.

#### 2.4. Data analysis

The quantitative data from the online questionnaire on previous dissemination activities were evaluated using SPSS^®^ Version 25 software [19].

For the evaluation of the qualitative interviews, the interpretative-reductive analysis was carried out as a combination of qualitative and quantitative analyzes. This organizes and categorizes the analyzed data in order to then work out relevant aspects. This allowed us to identify strong tendencies, new aspects and innovations in the interviews [13], [20], [21], [22], [23]. In order to maximally ensure the objectivity of the evaluation, independent raters also analyzed twelve randomly selected interviews using an intercoder reliability check and created categories based on their content [24].

#### 2.5. Ethics committee vote

The ethics committee of the Medical Faculty of the University of Ulm voted in favor for the quantitative research design on 16.06.2016 and for the qualitative research design on 20.09.2016.

## 3. Results

A total of 79 doctors took part in the online pilot course, 63 (79.7%) passed the course. The remaining 16 (20.3%) aborted the course prematurely (see figure 1 (Fig. 1)). The main reason for this was lack of time. The evaluation of the online course showed that 60.3% (n=38) of the doctors who completed the course had already disseminated course contents or materials. 88% (n=16) of the remaining 25 doctors who completed the course could imagine disseminating the course contents or materials in the future. The reasons for the dissemination of contents and materials were the need for further training among colleagues and the suitability of the course contents and materials. Dissemination was predominantly oral and took place in informal settings (see table 1 (Tab. 1)).

In the end it was possible to interview 25 of the 63 doctors who had completed the course. Table 2 (Tab. 2) shows the demographic characteristics of the interviewees.

The results of the interviews regarding motivation, obstacles and implementation possibilities for disseminating learning content from the online course by those who had completed it are presented below.

### 3.1. Awareness and meaning

The interviews showed that, according to the respondents, there is insufficient awareness of child protection in medicine. They stated that child protection must be promoted more in order to draw the necessary attention to the topic. The dissemination of knowledge can increase this awareness, since it goes beyond reading texts to exchanging ideas with colleagues. In addition, media coverage of the topic should also increase.

#### 3.2. Motivation to participating in a dissemination measure

The assessment of the relevance of the topic of child protection to the reality of one’s own work is said to have a great influence on the motivation for further training. Specialism, encountering an actual case and presence in the media are important factors for assessing the relevance of child protection.

Another motivation for doctors is gaining knowledge, e.g. about the processes, roles and networking partners in child protection, and thus confidence in their own actions. This alleviates fear of contact and other fears, e.g. making wrong decisions or falsely accusing someone and creates trust in one’s own capacities. In addition, this would also save time in the long term, which in turn would have a motivating effect.

 “And because a lot of ignorance also leads to looking away [...]. Or leads to not looking.”

/// Female doctor on the importance of knowledge in child protection 

Training points are also a strong motivation for doctors to continue their education.

#### 3.3. Motivation multipliers carrying out dissemination

The lack of time in the medical field is a well-known problem and is also important for potential multipliers. Release from work to prepare / carry out dissemination during working hours would motivate the interviewees to become involved as multipliers. The interviewees also mentioned payment or expense allowances as a possible incentive.

In addition, the relevance of the topic as motivation for engagement as a multiplier is important. Passing on knowledge about child protection in a group of colleagues and ultimately being able to help children was a great motivation for many of the interviewees. The fact that the range of further education courses in child protection in medicine is still very limited would further increase this intrinsic motivation.

“Meaningfulness.”

/// Male doctor on the question of what motivates him to disseminate contents on child protection.

As was already apparent in the quantitative survey, in the opinion of the interviewees the contents of the online course was suitable for dissemination but would have to be prepared accordingly prior to actual dissemination. Pre-prepared materials for this purpose would encourage motivation to carry out a dissemination measure. Such materials could be for face-to-face use such as presentation templates and exercises, but also handouts or posters. Instructions for implementation should be included with all materials, as the online course does not teach didactic dissemination skills.

#### 3.4. Obstacles to participation and carrying out a dissemination measure

Due to the lack of time, dissemination measure should be mandatory and designed to integrate into daily schedules of both participants and multipliers. Such activities counting as working hours or the inclusion of child protection in the specialization curriculum or in the existing training structures in everyday work would be one possibility. The participants felt it was also an obstacle that child protection was often not seen as a medical topic originally. The reason for this could be the often preventive nature of child protection, which does not correspond to the originally curative mandate of medicine.

“We only take action once something has actually happened to the child.”

/// Female doctor on awareness of child protection in medicine 

#### 3.5. Possibilities and ideas for implementing a dissemination measure

Practical and interactive methods are also important for the implementation of dissemination measures but each practical exercise should be preceded by a theoretical part. In addition to (hospital) internal training, the intranet was also mentioned as a dissemination vector which could provide information for all employees in everyday work.

The importance of a child protection expert for every medical workplace was also emphasized. These would then also be specially qualified to carry out appropriate dissemination measures.

With regard to the sustainability of dissemination measures, one idea that was brought up was to offer regular knowledge refreshers in addition to the dissemination of basic knowledge about child protection. Established structures such as newsletters or notices could also be used for such a purpose.

By repeatedly making it the subject of discussion. […] Maybe to tackle the topic twice, with an event as an intro and the second as a consolidation and then six months later, again and again, as a refresher.

/// Female doctor on sustainability in continuing education

## 4. Discussion

The aim of the online course “Child protection in medicine – a basic course for all health professionals” is to impart knowledge and capacities in the field of child protection to health professionals. The contents of the online course has already been evaluated positively and as relevant for everyday medical work. However, it was also shown that the range of continuing medical education courses on child protection in medicine is seen as being too small overall and that there is often not enough time to complete the online course [7]. In order to achieve a sustainable and comprehensive transfer of knowledge and capacity in child protection among medical staff – in addition to a general quantitative evaluation of the course – data on individual and detailed opinions on motivation, obstacles and implementation options in the area of active and targeted distribution (dissemination) of content from the online course through qualitative, semi-structured interviews with professionals who had completed the online course, were gathered and evaluated. In these interviews, those were asked to speak from the perspective of future multipliers.

It was shown that 60.3% of the doctors who had completed the course had already disseminated contents or materials from the online course due to the need for further training among colleagues and the suitability of the contents and materials. The qualitative interviews also made it clear that the dissemination of knowledge about child protection among health professionals is considered necessary by the respondents, although there are often concerns about the motivation for further training in this area. This is due, among other things, to the already recognized overload of health professionals [1], [2], [3]. Furthermore, a lack of knowledge can also lead to fear of contact with the topic. The level of awareness of child protection as a topic in medicine is still seen as being low.

However, the interviews also showed that various parameters play an important role in motivating doctors with regard to further training in general and dissemination of information in the area of child protection in particular. Based on this, conclusions can be drawn for different areas of the health care system.

### 4.1. Opportunities on the part of public institutions

Further and continuing education in the medical field in Germany is regulated by the Federal Medical Council, as the national committee above the regional medical councils [25]. The topic of child protection is currently only mandatory for specialization in pediatric and adolescent medicine, pediatric surgery, pediatric radiology and forensic medicine [26].

But since child abuse can cross into a wide range of specialist areas, doctors in other specializations should also have a basic knowledge of this area. Child protection must therefore be included in the continuing education curricula of all disciplines that work with children or adolescents. 

In addition, the responsibility of creating more jobs for specialists who are primarily dealing with child protection issues is the responsibility of the state. This was also confirmed by the recommendation of the German Medical Conference of 2017, which recommends the establishment of child protection groups in all facilities that care for children [27]. Increasing the presence of the topic in the media could also raise awareness of the issue.

#### 4.2. Opportunities on the part of in-patient and out-patient facilities

The results of the interviews confirmed that release from work, appropriate activities counting as working time or remuneration would motivate employees to offer or take part in dissemination measures. In addition, institutions should provide the opportunity to regularly participate in child protection training courses and on the current state of affairs. The intranet can also be made available as an exchange platform for contents and materials.

#### 4.3. Opportunities on the part of the multipliers

The dissemination of knowledge ultimately lies with the multipliers. It is essential to pass on the content as concisely as possible and to prioritize key aspects. The measures should also be designed in such a way that in the long term they lead to time savings in everyday work. This should be made clear before any event in order to increase the motivation of the target group.

A mix of theoretical and practical methods is also important when implementing the measures. Theoretical methods can be used to introduce and acquire basic knowledge, practical methods to acquire practical skills. Continuing Medical Education Points are also mentioned as a strong motivation for participation in training events. Appropriate certification of dissemination measures can be requested from the relevant regional medical councils.

Due to crowded schedules of health professionals, the underlying continuing education offer should provide ready-made dissemination materials.

#### 4.4. Limitations

In conclusion, it must be mentioned as a limiting factor that the interviewees represent only a selection of health professionals. Doctors are key actors in the field of continuing education of health professionals and the results are therefore a good basis for drawing further conclusions about other medical professions. It was not examined whether the results can be transferred to all doctors or to other medical areas. It can be assumed that especially those who have an increased interest in the topic of child protection and who rated the online course positively participated in the interviews. Since the aim of the investigation was to identify possibilities and obstacles to dissemination and not to evaluate the online course, the composition of the interviewees is appropriate. Furthermore, social desirability may be affecting the answers. In order to counteract this, it was made clear that the interviews were judgment-free, and the questions in the interview guide were worded openly in order to avoid influencing the answers. The data was pseudonymized or anonymized, this was communicated to the participants before the respective survey in order to avoid answers seen as socially desirable.

## 5. Conclusion

In summary, the interviewees report a high need and the importance of further training in child protection in medicine. It also shows a motivation to act as a multiplier and to carry out dissemination measures under certain conditions. The contents of the online course “Child protection in medicine - a basic course for all health professionals” is well suited to being disseminated. However, this contents should be converted into appropriate materials, such as presentation templates or exercises, in a way that allows the contents to be disseminated by multipliers as easily and effectively as possible.

## 6. Declarations

### 7.1. Ethics

A positive ethics committee vote of the medical faculty of the University of Ulm has been received. It does not constitute medical research on humans, therefore compliance with the ethical standards of the Helsinki Declaration and the Geneva Declaration is given.

#### 7.2. Author contributions

AM developed the research design, carried out the data collection, evaluated the results and wrote the manuscript. MR, UH and JMF gave assistance at all stages of the study and contributed to the interpretation of the data and the creation of the final manuscript. All authors have read and approved the final manuscript.

#### 7.3. Financial support

The project Development of an e-learning program “Child protection in medicine - a basic course for all health professionals” is funded by the Federal Ministry of Health as part of the “Promotion of Child Health” under the funding code [ZMVI1-2515KIG002]. Planning, conducting and evaluating the interviews and preparing the manuscript is part of the doctoral thesis by AM and therefore did not receive any financial support.

#### 7.4. Interview materials used

Maier A. Dissemination of the contents of the online course “Child protection in medicine - a basic course for all health professionals” [motivations, obstacles, implementation options and methods]. Telephone interview.

## Acknowledgements

Our thanks go to all 25 volunteers who made themselves available for an interview and who thus significantly supported the research project.

Mrs. Anne Straube and Mrs. Lena Preiss supported the work by carrying out the intercoder reliability check.

Our thanks also go to the “Birgit Maier Transcription Office” who transcribed the interviews.

## Competing interests

The authors declare that they have no competing interests. 

## Supplementary Material

Interview Guide

Method sheet

## Figures and Tables

**Table 1:**
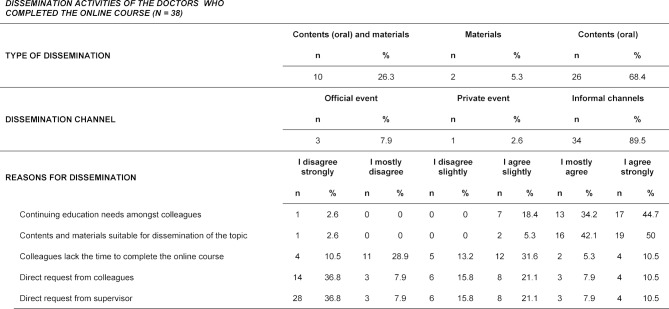
Dissemination activities of the doctors who completed the online course

**Table 2:**
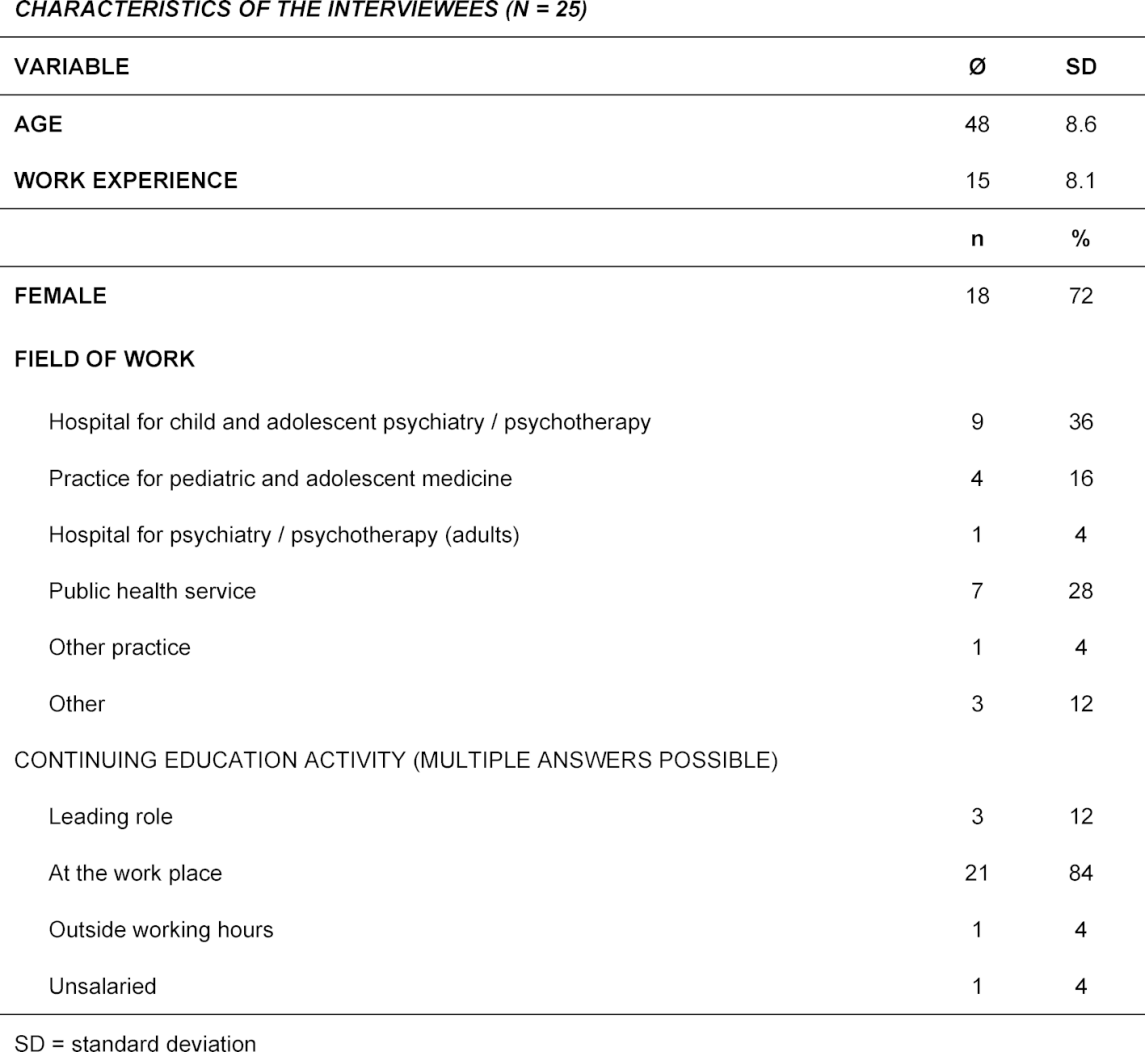
Characteristics of the interviewees.
