# Supplementary material for: Possibilities of dissemination of specialist knowledge and acting capacity in the field of child protection in medicine: a qualitative survey
Source: GMS J Med Educ. 2020 Feb 17;37(1):Doc10. doi: 10.3205/zma001303 (PMC7105763; doi:10.3205/zma001303)
Supplement: Interview Guide [file JME-37-10-s-001.pdf]

Name Date ID

Welcome and introduction

"Hello Mr/Mrs _____.

Glad to hear it worked. I already wrote to tell you that the interview will take about one hour all in all.

Let me briefly tell you about the background of the interview so that you will have an idea of what will happen to the data we are collecting.

The aim of this interview is to find out how the contents of the online course can be most effectively disseminated and which methods are suitable for dissemination, so that the standards of the continuing education topic of "Child protection in medicine" can be raised among medical staff over time. I would now like to ask you a few questions about your personal opinions and experience.

This survey is part of my doctoral thesis.

Please take your time in answering the questions, there are no right or wrong answers.

Any information you provide is anonymous and voluntary.

(Digital recording; if you wish to withdraw please let us know; it is possible to withdraw up to one week after the interview)

- *Is everything clear so far?*
- *Are you ready for the interview? Do you have any documents you might need at hand?"*

Guideline | Form

I. Demographic data

"For statistical purposes and so that I can better classify your professional background, I would like to collect some demographic data from you before we start the actual interview."

Current professional position: ○ employed — **Obligatory**

○ self-employed

○ on maternity or parental leave

○ unemployed

○ other __________________________________

Working as: ____ (job title) — **Obligatory**

[if not already mentioned] in which position: _______________________________ (especially if employed or on maternity / parental leave)	Optional
**Working in:** o own practice<br>o clinic / hospital<br>o employed in practice<br>o group practice<br>o other_____________________________________	**Obligatory**

II. Introduction

"Thank you for answering the first questions. Let us now start with the actual interview."

1. **How did you find out about the online course?**	**Obligatory (introductory question)**
2. **What would you do to reach and train as many people as possible on the topic of child protection in medicine? (Ideas / experiences)**	
3. **If you think back to the contents of the online course on Child Protection in Medicine and the materials used in it, do you think they are suitable for disseminating to colleagues? Explain why?**	**Obligatory (introductory question)**

III. Thematic block: Continuing education in the field of child protection in medicine

"I would now like to ask you a few questions about training and continuing education offers in the field of child protection in general"

1. **What is your view of the current range of continuing education offers on child protection in medicine? (Gaps → a. and b.; no gaps → c. and d.)** **Obligatory**

    a. You have just mentioned some gaps in the continuing education offers currently available. How do you think these gaps could be closed?

    b. In your opinion, what are the possibilities and limits of the online course in closing these gaps?

    c. In your opinion, what is the position of the online course in relation to these continuing education offers?

    d. What are the limits of the online course in your view?

2. Which continuing education offers in the field of child protection in medicine are you aware of, which ones do you use and how long do these offers take to complete? Optional

4. In your opinion, how big is the prevailing need among doctors for continuing education in general and how big is the need for continuing education in the field of child protection? **Optional (Priority 1)**

5. In your opinion, what is the prevailing motivation among doctors for continuing education in general and how great is the motivation for continuing education in the field of child protection? (Reasons) **Optional (Priority 1)**

IV. Thematic block: Dissemination of the learning contents of the online course

"Thank you for answering the first round of questions. The following is about your opinions on disseminating the learning contents of the online course to your colleagues after the course. It would be of interest if you could also consider your own workplace or work environment when answering the next questions."

1. **Have you passed on learning contents and materials to colleagues (yes → a.; no → b.)** **Obligatory**
   a. What exactly did you pass on and how did that take place?
   b. What were the reasons for not passing on any learning contents or materials?
2. **Do you have any ideas on how the learning contents of the online course could be passed on to colleagues in a targeted manner?** **Obligatory**
3. [If not yet mentioned] Where do you see difficulties and opportunities in passing on the learning contents of the online course? Optional
4. **In your view, how could colleagues be motivated to take part in continuing education by dissemination of the contents of the online course? (Incentives)** **Obligatory**
5. [If not mentioned] In your opinion, what structures are there in a medical work environment that could be used to disseminate the learning contents? (In your specific work environment) Optional

V. Thematic block: Working as a multiplier

"Finally, I would like to ask one very specific question. Imagine you were passing on the learning contents to your colleagues in the role of a Multiplier. Please answer the following questions with this in mind."

**1. Do you carry out training tasks at your workplace? (If yes → a)**	**Obligatory**
a. To what extent do you carry out training tasks?	Optional
**2. What would motivate you to work as a multiplier for the learning contents of the online course?**	**Obligatory**
3. What framework conditions would be important for you in your role as a multiplier?	Optional
**4. What materials could be used to support multiplier activity?**	**Obligatory**
**5. You received a short overview of various methods in advance. Imagine you were disseminating the learning contents to colleagues. Which methods do you think would be the most suitable for which contents and why?**<br>**a)** Do you have any other ideas etc. regarding this?	**Obligatory**

VI. Conclusion

**1. We have now reached the end of the interview. Are there other ways or methods you can think of for passing on what you have learned other than through multipliers; or is there anything else you would like to address regarding the online course and its dissemination?**	**Obligatory (final question)**

"Then please let me thank you again for your support. You have been a great help for my investigation. If you have any further questions about it or about using the interview materials, you are welcome to ask now or contact me afterwards."
