# Supplementary material for: Possibilities of dissemination of specialist knowledge and acting capacity in the field of child protection in medicine: a qualitative survey
Source: GMS J Med Educ. 2020 Feb 17;37(1):Doc10. doi: 10.3205/zma001303 (PMC7105763; doi:10.3205/zma001303)
Supplement: Method sheet [file JME-37-10-s-002.pdf]

Method overview for multipliers

You have agreed to take part in a qualitative telephone interview about the online course "Child protection in medicine - a basic course for all health professionals" and the dissemination of the course learning contents. The aim of the interview is to develop support offers, based on your feedback, for methods which will be made available to professionals who have completed the online course in the future.

To prepare for the interview, here is an overview of various methods that are used in the field of continuing education and can also be used for disseminating the online course. This overview should provide you with an introduction to the topic of adult education.

The methods are briefly explained in principle, followed by a description of what support offers provided by the project team for the method would look like. 'Materials' lists which additional equipment you need, 'Additional preparation' the things that have to be prepared in addition to engaging with the topic and the method.

Please read the overview, the different methods will be part of the interview. However, the aim of the interview will also be to capture your suggestions and ideas beyond these.

Thank you
The Project Team

© KJPP Ulm, University Hospital Ulm 2016

1. Methods for teaching theory[1,2]

The aim is to quickly convey theoretical subject matter and decision-making tools to determine possible courses of action. All methods require the multiplier to engage with the topic (text(s) on the learning content and, if necessary, further literature).

1.1 Presentation without visual aids

Method	Lecture on imparting knowledge
Implementation help	• Guide for the lecture<br>• Summary of the respective topics (can be used as a handout)
Materials	-
Additional preparation	Engaging with the lecture guidelines

1.2 Presentation with flipchart / poster

Method	Lecture with support / visualization by means of a poster or a designed flipchart, which can be used to explain and clarify the content.
Implementation help	Poster or template for a flipchart that can be developed during the lecture.
Materials	If necessary flipchart and flipchart marker (when using the flipchart)
Additional preparation	Engaging with the lecture guide and the poster / flipchart template

1.3 Presentation with PowerPoint / Prezi

Method	Lecture with support and visualization through PowerPoint slides / Prezi presentation[3].
Implementation help	• Content prepared in PowerPoint / Prezi presentation[4]<br>• Additional information for the lecture
Materials	Laptop and projector
Additional preparation	Engaging with the presentation

[1]https://www.m-c-p.ch/downloads/produktedaten/category/77-wolfvision-prospekte.html?download=105:wolfvision-5-praesentations-methoden
[2]http://www.wertarbeitgmbh.de/images/File/Methoden%20oder%20Weiterbildung.pdf
[3] Prezi presentations move through a larger overall picture and repeatedly pick up certain points in detail.
[4] Examples: https://prezi.com/de/gallery/

© KJPP Ulm, University Hospital Ulm 2016

1.4 Interactive presentation

Method	Lecture that is brought to life with interactive elements (e.g. filling out a diagram together with the participants). PowerPoint slides or a visualizer could be used as a medium.
Implementation help	• Guide for the lecture<br>• Summary of the respective topics<br>• Materials for joint development (e.g. worksheets, exercises)
Materials	Projector / visualizer
Additional preparation	Engaging with the materials to be developed

1.5 Group work

Method	The goal is self-directed group learning by solving a given task. The group work may be accompanied by a moderator.
Implementation help	• Different tasks for the group<br>• Stimulus questions that can be asked to stimulate the discussion
Materials	-
Additional preparation	Engaging with group tasks

© KJPP Ulm, University Hospital Ulm 2016

2. Practice-oriented methods[5,6,7]

The goal is to impart capacities for action and to learn from situations and experiences by engaging the participants. All methods first require theoretical engagement with the topic by the multiplier (text(s) on the learning content and, if necessary, further literature).

2.1 The 4-step method

Method	A method consisting of four learning stages. The aim is to reflect and expand the behavioral repertoire.<br>Preparation stage: Theoretical introduction, setting the learning goals and clarifying prior knowledge<br>Demonstration stage: Introduction to the scenario, demonstration and explanation of the correct procedure<br>Imitation stage: The procedure is mimicked and questioned by the participants until the participants are confident in their actions.<br>Final stage: Final consideration and evaluation
Implementation help	• Possible scenarios that can be processed using the 4 stages<br>• Instructions for the correct procedure<br>• Checklist with possible sources of errors, which require special attention<br>• Proposals for activating impulses in the simulation<br>• Proposals for the evaluation of the procedure
Materials	-
Additional preparation	Engaging with group tasks

2.2 Short simulation

Method	Simulation of a 3-10 minute work-related practice situation. The simulation is embedded in the theory, explained and discussed and evaluated after the presentation. The exercise tends to aim for improvisation.
Implementation help	• Specification of possible scenarios<br>• Checklist with possible sources of errors, which require special attention during the simulation<br>• Proposals for activating impulses in the simulation<br>• Proposals for the evaluation of the procedure.
Materials	-
Additional preparation	Engaging with group tasks

---

[5] based on http://www.wertarbeitgmbh.de/images/File/Methoden%20oder%20Weiterbildung.pdf
[6] Döring, Klaus w. & Ritter-Mamczek, Bettina: Lehren und Trainieren in der Weiterbildung – Ein praxisorientierter Leitfaden. BELTZ , 7. edition, Weinheim, 1999.
[7] Based on http://www.kollegiale-beratung.de/Ebene1/methode.html

© KJPP Ulm, University Hospital Ulm 2016

2.3 Role-play

Method	Precisely prepared working basis for a more elaborate simulation of more complex work situations. Role play has a preparation phase and a follow-up.
Implementation help	• Prepared and developed working basis<br>• Role descriptions for the participants<br>• Suggestions for evaluating role play
Materials	-
Additional preparation	Engaging with group tasks

2.4 Team supervision

Method	Form of advice that addresses with professional relationships. The goal is to improve professional practice. Cases from professional practice are discussed and processed together with other team members.
Implementation help	Case studies, if none arise from the participants' professional practice.
Materials	-
Additional preparation	Engaging with group tasks

2.5 Collegial advice based on the "Heilsbronner Model"

Method	Systematic counseling interview in which colleagues mutually advise each other and develop solutions together, based on the given discussion structure.[8]
Implementation help	Case studies, if none present themselves from the participants' professional practice.
Materials	-
Additional preparation	Engaging with group tasks

[8] http://www.rpz-heilsbronn.de/arbeitsbereiche/seelsorge-und-beratung/kollegiale-beratung.html

© KJPP Ulm, University Hospital Ulm 2016
